# Draft genome sequences of three xerophilic *Aspergillus* section *Restricti* species isolated from house dust in Japan

**DOI:** 10.1128/mra.00889-24

**Published:** 2024-12-16

**Authors:** Ryo Hagiuda, Dai Hirose

**Affiliations:** 1 School of Pharmacy, Nihon University, Funabashi, Chiba, Japan; University of Maryland School of Medicine, Baltimore, Maryland, USA

**Keywords:** *Aspergillus verrucosus*, food, indoor environment, phylogeny, taxonomy

## Abstract

We present the nuclear and complete mitochondrial genome sequences of *Aspergillus clavatophorus* NBRC 116038, *Aspergillus magnivesiculatus* NBRC 116037, and *Aspergillus verrucosus* NBRC 115547^T^, all isolated from house dust in Japan. These sequence data are crucial for elucidating the evolutionary characteristics of section *Restricti*, a unique taxon comprising exclusively xerophilic species.

## ANNOUNCEMENT

Xerophilic *Aspergillus* section *Restricti* species can survive in environments with low water activity (1). This taxon comprises 22 species classified into four series: *Halophilici*, *Penicillioides*, *Restricti*, and *Vitricolarum* (2–5). Recently, Hagiuda et al. (4) proposed a novel species of series *Halophilici*, *Aspergillus verrucosus*, isolated from house dust and honey in Japan. *Aspergillus verrucosus* should be a phylogenetically basal species in section *Restricti*; it should be a pivotal species for understanding the evolutionary process of this taxon. In this study, we present the draft genome sequences of *A. verrucosus* and two additional section *Restricti* species (*Aspergillus clavatophorus* and *Aspergillus magnivesiculatus*) that are frequently isolated from house dust in Japan. Phylogenetic analysis was performed using the obtained complete mitochondrial genome sequence.

Isolates were obtained from house dust in Aomori, Kanagawa, and Akita prefectures, Japan. The methods used for fungal isolation from house dust and species identification were described in detail by Hagiuda et al. (4, 6). Isolated strains were deposited in the Biological Resource Center, National Institute of Technology and Evaluation (NBRC), Chiba, Japan (Table 1).

**TABLE 1:** Summary of genome assembly and annotation for *Aspergillus clavatophorus* NBRC 116038, *Aspergillus magnivesiculatus* NBRC 116037, and *Aspergillus verrucosus* NBRC 115547^T^

Genome	Characteristic	Data for strain		
		*A. clavatophorus* NBRC 116038	*A. magnivesiculatus* NBRC 116037	*A. verrucosus* NBRC 115547^T^
Nuclear	No. of raw reads (M)	44.62	39.37	46.01
	Length of nuclear genome (Mb)	27.9	27.4	27.4
	No. of contigs	190	155	411
	Longest contig (bp)	895,710	910,384	774,262
	GC %	49.2	49	49.1
	N_50_ (bp)	255,737	396,436	141,380
	Sequencing depth (x)	137	379	281
	Genome completeness (%)	94.8	94.4	93.5
	No. of genes predicted using Funannotate	8,905	8,871	7,657
	No. of tRNAs	135	140	136
	No. of carbohydrate-active enzymes	319	313	279
	No. of regions identified using anti-SMASH	42	38	35
	No. of genes assigned and predicted using EggNOG	8,009	7,960	6,925
	DDBJ accession numbers	BAABNK010000000	BAABNL010000000	BAABNM010000000
Mitochondrial	Length of mitochondrial genome (bp)	45,861	33,008	139,059
	Genome completeness (%)	100	100	100
	GC %	25.4	25.8	27.3
	Protein-coding genes	13	13	13
	tRNAs	28	26	26
	rRNAs	2	2	2
	DDBJ accession number	LC797027	LC797028	LC797029

Genomic DNA was extracted from mycelia cultured on dichloran 18% glycerol agar (7) with a cellophane membrane for 14 days at 25°C using the benzyl chloride method (8). The concentration and purity of the extracted DNA was measured using a Nano Drop 1000 (Thermo Fisher Scientific, USA). A DNA library was prepared using the MGIEasy FS DNA Library Prep Set (MGI Tech, China) following the manufacturer’s protocol, and the DNBSEQ-G400 system (MGI Tech, China) was employed for genome sequencing with 2  ×  150 bp paired-end reads. Raw reads with adapters and low-quality reads were removed using FASTP ver. 0.20.1 (9). Sequence data processing steps used default parameters unless otherwise specified. *De novo* genome assembly was performed using MaSuRCA ver. 4 (10), with Pilon ver. 1.24 (11) was used twice to polish assembly errors. Genome completeness was assessed using Benchmarking Universal Single-Copy Orthologs program ver. 5.2.2 (12) with Eurotiales_odb10 data sets. Funannotate ver. 1.8.5 (13) was used to predict protein-coding genes in nuclear genomes and functional annotation was assigned using eggNOG-mapper ver. 2 (14) and HMMer ver. 3 (15) to search the eggNOG 5.0 database (16) and dbCAN3 web server (https://bcb.unl.edu/dbCAN2/) databases. In addition, genes involved in secondary metabolite production were identified using the antiSMASH web server (fungal ver. 7.0) (17). The mitochondrial genome was assembled separately using GetOrganelle ver. 1.7.6.1 (18). Assessment and manual editing of the assembly to obtain a complete circular contig was performed using Bandage ver. 0.8.171 (19). The mitochondrial genome was annotated using MITOS ver. 2 (20).

**Fig 1:**
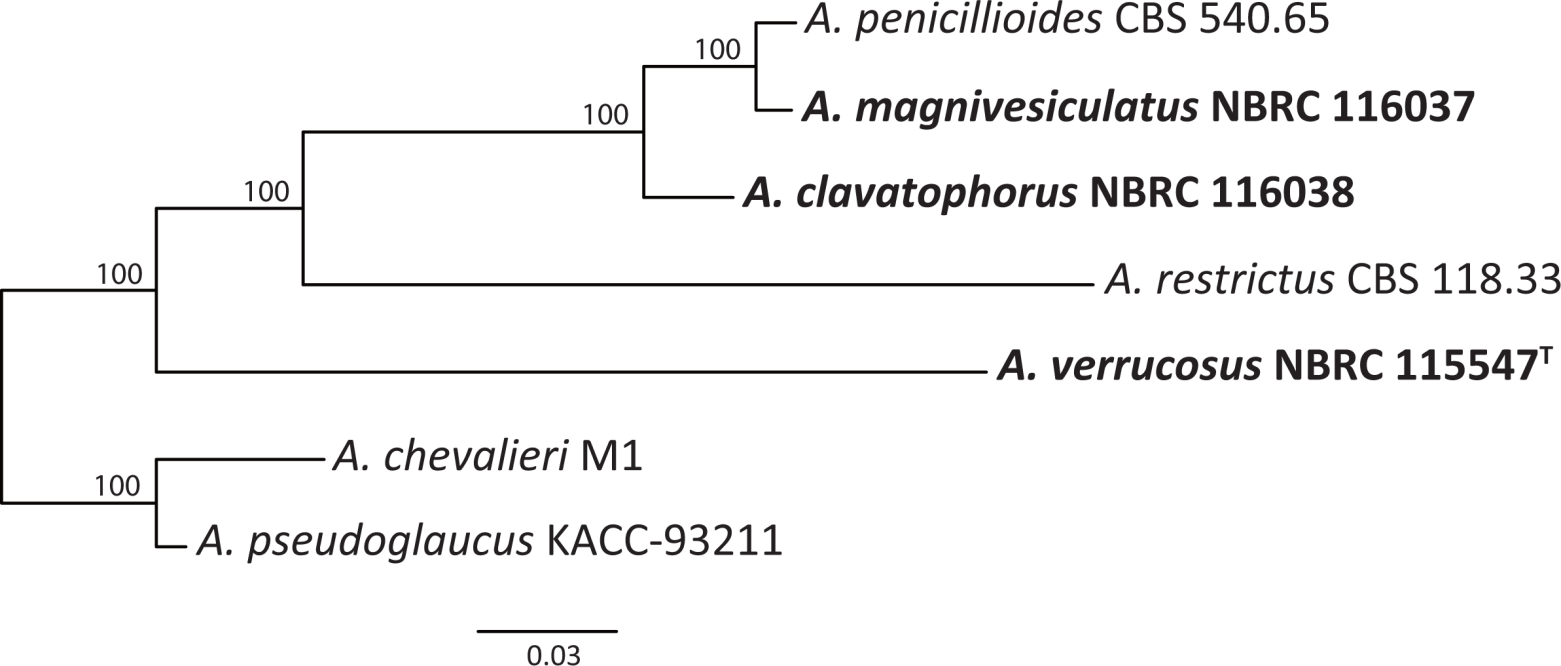
Maximum likelihood phylogenetic tree of *Aspergillus* section *Restricti* based on mitochondrial genome sequences. Mitochondrial genome sequences from *Aspergillus penicillioides* CBS 540.65 and *Aspergillus restrictus* CBS 118.33 (JGI: https://mycocosm.jgi.doe.gov/Aspergillus/Aspergillus.info.html) were used, along with newly obtained sequences. Following multiple alignments *via* MAFFT version 7 (21), phylogenetic analysis was performed using RAxML-NG version 1.0.1 (22) implemented in raxml GUI 2.0.5 (23). The nucleotide substitution model TIM1+I was selected using Model Test-NG version 0.1.6 (24). Bootstrap support for each clade, calculated using the bootstrap method of Lemoine et al. (25), is shown above the branches. *Aspergillus chevalieri* M1 (GenBank: AP024424) and *Aspergillus pseudoglaucus* KACC-93211 (GenBank: MK202802) were chosen as outgroups.

Genome assembly and annotation statistics for three isolates are shown in Table 1. Based on mitochondrial genome sequences, maximum likelihood phylogenetic analysis supported the monophyly of species other than *A. verrucosus* (bootstrap value = 100), suggesting that this species is the basal lineage in section *Restricti* (Fig. 1). The obtained genome sequence data will be crucial for comparative genomics that may elucidate the evolutionary characteristics of the xerophilic section in the primarily nonxerophilic genus *Aspergillus*.

## Data Availability

The project data of *A. clavatophorus* NBRC 116038, *A. magnivesiculatus* NBRC 116037, and *A. verrucosus* NBRC 115547^T^ were deposited at the DDBJ/ENA/GenBank as BioProject accession number PRJDB17030, BioSample accession numbers SAMD00657600, SAMD00657601, and SAMD00657602, respectively, and SRA accession numbers DRX514604, DRX514605, and DRX514606, respectively. Accession numbers for each isolate genome are listed in Table 1. This announcement reports the first versions of these genome sequences.
